# LORSEN: Fast and Efficient eQTL Mapping With Low Rank Penalized Regression

**DOI:** 10.3389/fgene.2021.690926

**Published:** 2021-11-17

**Authors:** Cheng Gao, Hairong Wei, Kui Zhang

**Affiliations:** ^1^ Department of Mathematical Sciences, Michigan Technological University, Houghton, MI, United States; ^2^ College of Forest Resources and Environmental Science, Michigan Technological University, Houghton, MI, United States

**Keywords:** eQTL mapping, proximal gradient method, cis-eQTL, *trans*-eQTL, penalized regression

## Abstract

Characterization of genetic variations that are associated with gene expression levels is essential to understand cellular mechanisms that underline human complex traits. Expression quantitative trait loci (eQTL) mapping attempts to identify genetic variants, such as single nucleotide polymorphisms (SNPs), that affect the expression of one or more genes. With the availability of a large volume of gene expression data, it is necessary and important to develop fast and efficient statistical and computational methods to perform eQTL mapping for such large scale data. In this paper, we proposed a new method, the low rank penalized regression method (LORSEN), for eQTL mapping. We evaluated and compared the performance of LORSEN with two existing methods for eQTL mapping using extensive simulations as well as real data from the HapMap3 project. Simulation studies showed that our method outperformed two commonly used methods for eQTL mapping, LORS and FastLORS, in many scenarios in terms of area under the curve (AUC). We illustrated the usefulness of our method by applying it to SNP variants data and gene expression levels on four chromosomes from the HapMap3 Project.

## 1 Introduction

With rapid advancements in sequencing technologies and high-throughput technologies, a large number of single nucleotide polymorphism (SNP) data and gene expression data have become available. This allows us to investigate the associations between SNP genotypes and gene expression levels. Expression quantitative trait loci (eQTLs) are those genetic variants that can explain variation in gene expression levels and can help to elucidate the underlying genetic mechanisms of human complex traits (Albert and Kruglyak, 2015). eQTL mapping aims to identify eQTLs associated with genes of interest (Hu et al., 2015; Banerjee et al., 2021). In general, eQTLs are classified into two types: *cis*-eQTLs (or local eQTLs) and *trans*-eQTLs (or distant eQTLs) (Cookson et al., 2009). *cis*-eQTLs refer to the genetic variants that functionally act on local genes and are physically located close to the target genes. *trans*-eQTLs are those genetic variants that functionally act on distant genes residing on the same or different chromosome and are physically located far from the target genes. It is worth mentioning that *trans*-eQTLs account for a large proportion of heritability of gene expression levels, though *trans* effects are usually weaker than *cis* effects in humans (Cookson et al., 2009).

In fact, gene expression levels observed are not only regulated by genetic variants but also influenced by non-genetic factors which are known or hidden, for example, batch effects. Therefore, in eQTL mapping, how to account for confounding factors is an important issue and can influence the detection power of eQTL mapping. Up to now, a number of methods have been proposed to account for confounding factors in eQTL mapping, for example, PANAMA (Fusi et al., 2012), PEER (Stegle et al., 2010), LORS (Yang et al., 2013), HEFT (Gao et al., 2014), LMM-EH-PS (Listgarten et al., 2010) and ECCO (Yue et al., 2020). Another challenge in eQTL mapping is that the number of SNPs involved is usually very large (Yang et al., 2013). This not only results in heavy computational burden for estimating model parameters but also generally results in reduced detection power if all SNPs are included in eQTL mapping. This is because the signal-to-noise ratio (SNR) is very low, meaning only a very small portion of SNPs that are actually associated with gene expression levels. To overcome this problem, a number of SNP screening procedures (Wang et al., 2011; Yang et al., 2013; Jeng et al., 2020) and variable selection techniques (Fan and Lv, 2008) that aim to reduce the number of SNPs and only keep informative SNPs in eQTL mapping have been developed. More importantly, a number of methods based on the penalized regression have been developed to model such sparsity of eQTLs (Lee and Xing, 2012; Yang et al., 2013; Cheng et al., 2014; Jeng et al., 2020).

LORS, a method based on the low rank sparse regression, was proposed for eQTL mapping in (Yang et al., 2013). LORS is based on a linear model with gene expression levels as response variables and SNP genotypes as predictors. To model the sparsity of regression coefficients, LORS poses the *L*_1_ penalty on the regression coefficient matrix. In addition, LORS includes one unknown matrix with the nuclear norm penalty to account for variations caused by non-genetic factors. Yang et al. (2013) applied the coordinate descent algorithm to optimize the objective function and estimate the model parameters. A SNP screening method, called LORS-Screening, was also developed to reduce the number of SNPs involved in the subsequent joint modeling, thus reduce the computational burden greatly. Similar to LORS, FastLORS (Jeng et al., 2020) employs the same low rank sparse regression model that is used in LORS. Different from LORS, FastLORS uses generic proximal gradient algorithm to optimize the objective function and estimate the model parameters. Moreover, Jeng et al. (2020) proposed a SNP screening method based on the Higher Criticism (HC) statistic, called HC-Screening.

To improve the detection power of eQTL mapping, a number of methods have been developed to incorporate the structure information from SNP variants data and gene expression levels, for example, clustering based on gene expression levels (Kendziorski et al., 2006; Chun and Keles, 2009) and gene regulatory networks (Rakitsch and Stegle, 2016), into eQTL mapping. A number of studies have shown that such structure information from SNP variants data and gene expression levels can be effectively used in penalized regression to boost the detection power of eQTL mapping (Chen et al., 2012; Kim and Xing, 2012, 2009). For example, the graph-regularized dual lasso (GDL) proposed by (Cheng et al., 2014) can simultaneously integrate the correlation structures among SNPs and gene expression levels. Through extensive experimental evaluations, Cheng et al. (2014) showed that GDL significantly outperformed the existing method for eQTL mapping. Similar to GDL, the graph-guided fused lasso (GFlasso) proposed by (Lee and Xing, 2012) can also consider the structure of the genetic variants and the structure of the gene expression levels. As a penalized regression method, GFlasso also inherits the benefits from the group lasso. Lee and Xing (2012) showed that GFlasso was able to detect weak association signals between the genetic variants and the gene expression levels.

However, there are some drawbacks for most of the aforementioned methods. First, if two SNPs are highly correlated with each other, and one SNP is associated with some genes, but the other SNP is not associated with them, we should not expect that these two SNPs have similar coefficients for those genes. Similarly, if some SNPs are classified into one group, we should not expect that the SNPs within the same group have similar coefficients for common genes. Second, the group structures of SNP data and gene expression data are usually identified by performing clustering on the data, however, clustering is an unsupervised leaning approach, the number of clusters is usually artificially determined. When we use the resulting clusters of SNPs and gene expressions to design the penalty term, it may lead to loss of detection power and even spurious associations. Third, complicated design of penalty term in penalized regression modeling can result in untractable computational bottleneck, especially when dealing with a large volume of data.

To overcome such limitations of existing methods for eQTL mapping, we proposed a novel method, LOw Rank Sparse regression with Elastic Net penalty, abbreviated as LORSEN. Different from LORS (Yang et al., 2013) and FastLORS (Jeng et al., 2020), we applied the Elastic Net penalty to the association coefficients instead of the *L*_1_ penalty in LORSEN. In addition, we used the low rank approximation to account for non-genetic factors in LORSEN (Yang et al., 2013). There are several advantages to use the Elastic Net penalty instead of the *L*_1_ penalty (Tibshirani, 1996). First, when the number of SNPs *p* is much larger than the sample size *n*, theoretically, the methods based on the *L*_1_ penalty can only yield at most *n* non-zero coefficients. This can lead to the substantial loss of detection power in eQTL mapping since the number of samples is generally much smaller than the number of eQTLs in gene expression studies. Second, when several eQTLs are in linkage disequilibrium (LD), the methods based on the *L*_1_ penalty can only select one of them. In theory, the Elastic Net penalty can overcome these two drawbacks. For the estimation of the model parameters in LORSEN, we developed an efficient optimization algorithm based on the proximal gradient method (Parikh and Boyd, 2014). Our algorithm allows us to perform the eQTL mapping for a large number of SNPs and genes. We evaluated and compared the performance of LORSEN with LORS and FastLORS using extensive simulation studies as well as the HapMap3 data.

## 2 Material and Methods

### 2.1 Model

We assume that the genotypes for *p* SNPs and the gene expression levels for *q* genes over *n* samples are collected. Let *X* denote the *n* × *p* matrix of SNP genotypes coded in an additive manner, and *Y* denote the *n* × *q* matrix of gene expression levels. To model the association between SNPs and gene expressions, we can use the following multivariate linear model as proposed in (Yang et al., 2013):

$$Y=XB+L+1\mu^{T}+e,$$ (1)

where *B* is a *p* × *q* matrix for the regression coefficients, **1** is a *n*-dimensional all-ones vector, *μ* is a *q*-dimensional vector for the intercepts in the regression model, *e* is a *n* × *q* matrix for the error terms and each element in *e* has a normal distribution with zero mean and variance *σ* ^2^, all *e*_*ij*_ are independent, *L* is a *n* × *q* matrix which is introduced to account for variations caused by non-genetic factors.

For the convenience of description, we first introduce the following notations used in this paper. For a *n*-dimensional vector *v* with the elements *v*_*i*_(*i* = 1, …, *n*): the *L*_1_ norm of *v* is defined as $\left\|v{\|}_{1}=\Sigma_{i=1}^{n}|v_{i}\right|$ (the sum of absolute values of the elements) and the *L*_2_ norm (also called the Euclidean norm) of *v* is defined as $\|v{\|}_{2}=\sqrt{\Sigma_{i=1}^{n}v_{i}^{2}}$ (the squared root of the sum of squares of the elements), respectively. For a *m* × *n* matrix *M* with the elements *M*_*ij*_(*i* = 1, …, *m*; *j* = 1, …, *n*), the Frobenius norm of *M* is defined as $\|M{\|}_{F}=\sqrt{\Sigma_{i=1}^{m}\Sigma_{j=1}^{n}M_{ij}^{2}}$ (the squared root of the sum of squares of the elements); the nuclear norm $\|M{\|}_{*}=\Sigma_{i=1}^{r}\sigma_{i}$ , where *σ*_1_, …, *σ*_*r*_ are the singular values of *M* and *r* is the rank of *M*; and the *L*_1_ norm of *M* is defined as $\left\|M{\|}_{1}=\Sigma_{i=1}^{m}\Sigma_{j=1}^{n}|M_{ij}\right|$ (the sum of absolute values of the elements).

In this paper, we follow the same sparsity assumptions used in (Yang et al., 2013). First, we assume that there are only a small number of non-genetic factors that influence the gene expression levels globally, not locally. Second, we assume that there are only a small fraction of SNPs that influence the gene expression levels. This assumption implies that the regression coefficient matrix *B* is sparse. Yang et al. (2013) proposed the following LORS procedure to estimate *B*, *L*, *μ* by solving the optimization problem

$$\underset{B,L,\mu }{\min}\;\frac{1}{2}\|Y-XB-L-1\mu^{T}{\|}_{F}^{2}+\rho \|L{\|}_{*}+\lambda \|B{\|}_{1},$$ (2)

where *ρ* and *λ* are regularization (tuning) parameters that control the rank of *L* and the sparsity of *B*, respectively. When *L* and *μ* are fixed, the optimization problem becomes a least absolute shrinkage and selection operator (Lasso) (Tibshirani, 1996) problem with respect to *B*. As pointed out in (Zou and Hastie, 2005), the Lasso has some limitations that affect its usefulness. First, when *n* < *p* (the number of samples is smaller than the number of SNPs), the Lasso selects at most *n* SNPs. In the context of eQTL mapping, there are usually a small number of samples available. Even though the proportion of SNPs that are associated with the gene expression levels is small, it is highly likely that the number of SNPs associated with the gene expressions can still be larger than the number of samples. In this case, the *L*_1_ penalty on *B* will fail to identify some SNPs that are associated with the gene expressions. Second, the Lasso tends to select only one variable among a group of highly correlated variables. This can be problematic in eQTL mapping. For example, if two SNPs are in high linkage disequilibrium and both of them are associated with gene expressions, only one SNP will be selected by the Lasso. Furthermore, if two SNPs are in high linkage disequilibrium and only one of them is associated with gene expressions, the selected SNP by the Lasso may not even be associated with gene expressions.

The use of the Elastic Net penalty (Zou and Hastie, 2005) instead of the *L*_1_ penalty on *B* can overcome the limitations of the Lasso. Therefore, we propose the following optimization problem to estimate *B*, *L*, *μ*:

$$\underset{B,L,\mu }{\min}\;\frac{1}{2}\|Y-XB-L-1\mu^{T}{\|}_{F}^{2}+\rho \|L{\|}_{*}+\lambda_{1}\|B{\|}_{1}+\frac{\lambda_{2}}{2}\|B{\|}_{F}^{2},$$ (3)

where *ρ*, *λ*_1_ and *λ*_2_ are non-negative tuning parameters. For real data sets, it is quite possible that some entries in *Y* are unobserved (missing). In such scenarios, the missing data will not be used in (Eq. 3). As used in (Yang et al., 2013), we use Ω to index the observed entries in *Y*. Specifically, Ω is a *n* × *q* matrix with the entry

$$\Omega_{ij}=\left\{\begin{array}{c} 0,\;Y_{ij}\;missing\;\\ 1,\;otherwise.\; \end{array}\right.$$ (4)

Then we define the projection of a matrix *A* onto Ω as $\tilde{A}=P_{\Omega }\left(A\right)=\Omega ⊚A$ , where A has the same dimension as Ω and ⊚ represents Hadamard product, that is, ${\tilde{A}}_{ij}=A_{ij}\times \Omega_{ij}$ . Based on the observed data, the optimization problem becomes

$$\underset{B,L,\mu }{\min}\;\frac{1}{2}\|P_{\Omega }\left(Y-XB-L-1\mu^{T}\right){\|}_{F}^{2}+\rho \|L{\|}_{*}+\lambda_{1}\|B{\|}_{1}+\frac{\lambda_{2}}{2}\|B{\|}_{F}^{2}.$$ (5)

### 2.2 Theory and Algorithm

To solve the optimization problem in (Eq. 5) efficiently, we developed a fast and efficient algorithm based on proximal gradient method (Parikh and Boyd, 2014).

We first describe the proximal gradient method for a general optimization problem

$$\underset{x}{\min}\;f\left(x\right)=g\left(x\right)+h\left(x\right),$$ (6)

where *g*(*x*) is a convex and differentiable function, *h*(*x*) is a closed proper convex which means *h*(*x*) is a convex function, the epigraph of *h*(*x*) is closed and *h*(*x*) < +*∞* for at least one *x* and *h*(*x*) > −*∞* for every *x*. Furthermore, we assume that ∇*g*(*x*), the gradient of *g*(*x*), is Lipschitz continuous with constant *ℓ*, which implies that ∇^2^ *g*(*x*)⪯*ℓ* **I**. Two symmetric matrices of the same dimensions *A* and *B* have the relationship *A*⪯*B*, if *B* − *A* is positive semidefinite. Then we have

$$f\left(x\right)=g\left(x\right)+h\left(x\right)⩽g\left(x_{0}\right)+\left\langle \nabla g\left(x_{0}\right),x-x_{0}\right\rangle +\frac{1}{2t}\|x-x_{0}{\|}^{2}+h\left(x\right),\;t\in \left(0,\frac{1}{\ell }\right],$$ (7)

where *x*_0_ is an arbitrary point in the domain of *f*(*x*) and ⟨⋅, ⋅⟩ represents the inner product of two vectors. Instead of using the optimization problem (Eq. 6), we focus on minimizing an upper bound of the objective function, that is,

$$\underset{x}{\min}\;g\left(x_{0}\right)+\left\langle \nabla g\left(x_{0}\right),x-x_{0}\right\rangle +\frac{1}{2t}\|x-x_{0}{\|}^{2}+h\left(x\right),\;t\in \left(0,\frac{1}{\ell }\right],$$ (8)

which can be interpreted as an application of majorization-minimization algorithm (Parikh and Boyd, 2014). The optimization problem in (Eq. 8) is equivalent to the following optimization problem:

$$\underset{x}{\min}\;\frac{1}{2t}\|x-\left(x_{0}-t\nabla g\left(x_{0}\right)\right){\|}^{2}+h\left(x\right).$$ (9)

Problem (Eq. 9) can be solved with an iterative procedure: given the value of *x* at the *k*-th iteration, i.e., *x*_*k*_, the value of *x* at the *k* + 1-th iteration, *x*_*k*+1_ can be updated by the following formula

$$x_{k+1}=\underset{x}{\mathrm{arg min}}\;\frac{1}{2t}\|x-\left(x_{k}-t\nabla g\left(x_{k}\right)\right){\|}^{2}+h\left(x\right)=Prox_{t,h}\left(x_{k}-t\nabla g\left(x_{k}\right)\right),$$

where *Prox*(⋅) is called proximal operator. The iterative process is repeated until the stopping criterion is satisfied or the maximum number of iterations is reached.

To solve the optimization problem (Eq. 5), we adopted an alternating optimization approach that is similar to the method in (Yang et al., 2013). Note that in the following part, *t*_*L*_, *t*_*B*_, and *t*_*μ*_ are like *t* used in problem (Eq. 9) and correspond to the variables *L*, *B*, and *μ*, respectively.

First, for fixed *B* and *μ*, (Eq. 5) becomes

$$\underset{L}{\min}\;\frac{1}{2}\|Y-XB-1\mu^{T}-L{\|}_{F}^{2}+\rho \|L{\|}_{*}.$$ (10)

In the setting of optimization problem (Eq. 10), $\frac{1}{2}\|Y-XB-1\mu^{T}-L{\|}_{F}^{2}$ plays the role of *g*(*x*) and *ρ*‖*L*‖_*_ plays the role of *h*(*x*) in (Eq. 6). By Lemma 1 (Appendix A), at the *k* + 1-th iteration, we have

$$\begin{array}{ll} L_{k+1} & =Prox_{t_{L},\rho \|\cdot {\|}_{*}}\left(L_{k}-t_{L}\left(XB_{k}+1\mu_{k}^{T}+L_{k}-Y\right)\right)\\ & =S_{t_{L}\rho }\left(L_{k}-t_{L}\left(XB_{k}+1\mu_{k}^{T}+L_{k}-Y\right)\right), \end{array}$$

where $S_{t_{L}\rho }\left(\cdot \right)$ is the singular value shrinkage operator (please refer to the Appendix A), *t*_*L*_ is the step size which can be constant or be determined by backtracking line search.

Second, for fixed *L* and *μ*, then (Eq. 5) becomes

$$\underset{B}{\min}\;\frac{1}{2}\|Y-XB-L-1\mu^{T}{\|}_{F}^{2}+\lambda_{1}\|B{\|}_{1}+\frac{\lambda_{2}}{2}\|B{\|}_{F}^{2},$$ (11)

where *t*_*B*_ is the step size which can be constant or be determined by backtracking line search. By Lemmas 2 and 3 and Theorem 1 (Appendix A), we can update *B*_*k*+1_ accordingly:

$$\begin{array}{ll} B_{k+1}^{a} & =B_{k}-t_{B}X^{T}\left(XB_{k}+1\mu_{k}^{T}+L_{k+1}-Y\right)\\ B_{k+1}^{b} & =Prox_{t_{B},\lambda_{1}\|\cdot {\|}_{1}}\left(B_{k+1}^{a}\right)\\ & =sign\left(B_{k+1}^{a}\right)⊚{\left(|B_{k+1}^{a}|-\lambda_{1}J\right)}_{+}\\ B_{k+1}\left[,j\right] & =Prox_{t_{B},\lambda_{2}\|\cdot {\|}_{2}}\left(B_{k+1}^{b}\left[,j\right]\right)\\ & =\left\{1-\frac{\lambda_{2}}{max\left\{\|B_{k+1}^{b}\left[,j\right]{\|}_{2},\lambda_{2}\right\}}\right\}B_{k+1}^{b}\left[,j\right],\;j=1,2,\ldots ,q, \end{array}$$

where *J* is a all-ones *p* × *q* matrix, *B*[, *j*] is the *j*-th column of matrix *B* and is a *p*-dimensional vector, *γ*_+_ =  max{*γ*, 0}, the maximum of *γ* and 0, $\left|B_{k+1}^{a}\right|$ , $\mathrm{sign}\left(B_{k+1}^{a}\right)$ , and ${\left(|B_{k+1}^{a}|-\lambda_{1}J\right)}_{+}$ are all elementwise operations.

Third, for fixed *L* and *B*, the proximal gradient method reduces to the gradient descent method with respect to *μ* because there is no penalty on *μ*. At the *k* + 1-th iteration, we have

$$\mu_{k+1}=\mu_{k}-t_{\mu }{\left(XB_{k+1}+1\mu_{k}^{T}+L_{k+1}-Y\right)}^{T}1.$$

To accelerate the computational speed, we used the accelerated proximal gradient method. Specifically, we applied the fast iterative shrinkage-thresholding algorithm (FISTA) (Beck and Teboulle, 2009) which keeps the simplicity of the iterative shrinkage-thresholding algorithms (ISTA) but has an improved rate *O*(1/*k* ^2^), where *k* indexes the iteration. In FISTA, the step size can be constant or be determined by backtracking line search. The algorithm to solve LORSEN with FISTA is described in Algorithm 1. For simplicity, here, only the detailed algorithm with the constant step size is described, but the algorithm using the step size determined by backtracking line search is also provided in our R program (https://github.com/gaochengPRC/LORSEN).

### 2.3 Parameter Tuning

For parameter tuning, we mainly followed the idea described in (Yang et al., 2013). Specifically, we divided the entries of Ω into training entries and testing entries such that training entries and testing entries include roughly the same number of 1’s. We define two matrices Ω_1_ and Ω_2_ such that they have the same dimensions as Ω, Ω_1_ contains all training entries and Ω_2_ contains all testing entries. Furthermore, we have Ω = Ω_1_ + Ω_2_ and Ω_1_ ⊚Ω_2_ = 0. For the consistency, we re-parameterized *λ*_1_ and *λ*_2_ as *λ* ⋅ *α* and *λ* ⋅ (1 − *α*), respectively. So the optimization problem (Eq. 5) becomes

$$\underset{B,L,\mu }{min}\;\frac{1}{2}\|Y-XB-L-1\mu^{T}{\|}_{F}^{2}+\rho \|L{\|}_{*}+\lambda \left(\alpha \|B{\|}_{1}+\frac{1-\alpha }{2}\|B{\|}_{F}^{2}\right).$$ (12)

This form is the same as that in glmnet (Friedman et al., 2010).

Given the values of parameters (*ρ*, *α*, *λ*), we solve the following optimization problem

$$\underset{B,L,\mu }{min}\;\frac{1}{2}\|P_{\Omega_{1}}\left(Y-XB-L-1\mu^{T}\right){\|}_{F}^{2}+\rho \|L{\|}_{*}+\lambda \left(\alpha \|B{\|}_{1}+\frac{1-\alpha }{2}\|B{\|}_{F}^{2}\right).$$ (13)

The solutions are *B*(*ρ*, *α*, *λ*), *L*(*ρ*, *α*, *λ*) and *μ*(*ρ*, *α*, *λ*), then we evaluate the parameters by calculating the prediction error

$$Err\left(\rho ,\alpha ,\lambda \right)=\frac{1}{2}\|P_{\Omega_{2}}\left(Y-XB\left(\rho ,\alpha ,\lambda \right)-L\left(\rho ,\alpha ,\lambda \right)-1\mu {\left(\rho ,\alpha ,\lambda \right)}^{T}\right){\|}_{F}^{2}.$$ (14)

The grid search over three parameters may be too computationally intensive. Therefore, we first found an optimal value for *ρ*, $\hat{\rho }$ , which minimizes the prediction error as shown in (Yang et al., 2013) by means of Lemmas 1 and 4 (Appendix A). Please refer to (Yang et al., 2013) to find the details about how to find the optimal value of *ρ*, $\hat{\rho }$ . Once the optimal value of *ρ*, $\hat{\rho }$ is obtained, we selected a value of *α* from a sequence sequentially, thereafter, we performed one-dimensional grid search for *λ* for each *α*. Specifically, we generated a sequence of *λ* values with length *n*_*λ*_ decreasing from $\lambda_{max}\left(\hat{\rho },\alpha \right)$ to $\epsilon \lambda_{max}\left(\hat{\rho },\alpha \right)$ on the log scale with equal space, where $\lambda_{max}\left(\hat{\rho },\alpha \right)$ is defined as the smallest *λ* such that $B\left(\hat{\rho },\alpha ,\lambda \left(\hat{\rho },\alpha \right)\right)$ is a zero matrix. $\lambda_{max}\left(\hat{\rho },\alpha \right)$ is derived as $\frac{1}{\alpha }\underset{i=1,2,\ldots ,p}{max}\;\underset{j=1,2,\ldots ,q}{max}|\left\langle X_{i},Y_{j}\right\rangle |$ from coordinate-descent algorithm (Friedman et al., 2007), where *X*_*i*_ is the *i*-th column of *X*, and *Y*_*j*_ the *j*-th column of *Y*. In our R program, we set *ϵ* = 0.02, *n*_*λ*_ = 50 and *S*_*α*_≔(0.2, 0.4, 0.6, 0.8, 0.9). The optimal parameters were $\left(\hat{\rho },\alpha ,\hat{\lambda }\left(\hat{\rho },\alpha \right)\right)$ that minimize the prediction error. The optimal feasible solutions of *B*, *L*, and *μ* were then obtained based on the set of optimal tuning parameters.

### 2.4 Single Nucleotide Polymorphism Ranking and Joint Modeling

The procedure to select the set of optimal tuning parameters is computationally intensive. Therefore, as it is discussed in (Yang et al., 2013), it may not be computationally tractable to directly apply such method to the large-scale data sets that contain a large number of gene expression levels and SNPs. A commonly used strategy to reduce such computational burden is to choose a subset of SNPs and then only use them in the subsequent eQTL analysis. In this paper, we used and evaluated two existing methods for the pre-selection of informative SNPs: LORS-Screening (Yang et al., 2013) and Higher Criticism Screening (HC-Screening) (Jeng et al., 2020). For LORS-Screening, we first obtained the initial estimate of *β*_*i*_’s by solving

$$\underset{\beta_{i},L,\mu }{min}\;\frac{1}{2}\|Y-X_{i}\beta_{i}^{T}-L-1\mu^{T}{\|}_{F}^{2}+\rho \|L{\|}_{*},$$ (15)

where *X*_*i*_ is the *i*-th column of *X*, *β*_*i*_ is a *q*-dimensional vector for the coefficient of the *i*-th SNP on *q* genes, *i* = 1, 2, …, *p*. For each gene, we selected the top *n* SNPs in terms of the absolute values of association coefficients, then we obtained the union of selected SNPs for each gene as the final set of SNPs to be involved in the joint modeling. For HC-Screening, we first obtained association coefficients as above, then calculated the standardized estimates of coefficients. For each SNP, the Higher Criticism (HC) statistic (Donoho and Jin, 2004) is calculated based on the standardized estimates of coefficients. Then we selected the top *n* SNPs in terms of the *p*-values of HC statistics.

### 2.5 Simulation Design

Our simulation is similar to that described in (Jeng et al., 2020). We first downloaded the genotype data of Chromosome 1 and Chromosome 21 for CEU samples from HapMap3, the third phase of the International HapMap Project (https://www.genome.gov/10001688/international-hapmap-project). CEU samples refer to Utah residents with Northern and Western European ancestry from the CEPH collection. After the quality-control (please refer to Real Data Analysis section), the genotype data of 13,815 SNPs of Chromosome 1 and 2,607 SNPs of Chromosome 21 for *n* = 165 samples were retained in analysis. To simulate gene expression levels for *q* = 200 genes over *n* = 165 samples, we first simulated non-genetic effects of *k* = 15 hidden factors. We randomly generated *nk* random numbers from *N*(0, 1) to form a *n* × *k* matrix *H*, then let Σ = *HH* ^*T*^. *U*_*j*_’s were simulated from *N*(0, 0.1*Σ), *j* = 1, 2, …, *q* and stacked by column to form a *n* × *q* matrix *U*. *e*_*j*_’s were simulated from *N*(0, *I*) as random noise for *j*-th gene expression and combined by column to form a *n* × *q* random noise matrix *e*. Then the expression data of *q* genes over *n* samples were simulated by *Y* = *XB* + *U* + *e*, where *X* is the *n* × *p* genotype data matrix. We set the total number of SNPs *p* = 2000, the number of causal SNPs as 60, 200, or 400. Each causal SNP randomly influences *m* = 10 (or 50) genes. We simulated nonzero genetic effects from a uniform distribution. For the “weak-dense” scenario, each causal SNP affects *m* = 50 randomly selected genes and the corresponding values in *B* were simulated from a uniform distribution between 0.25 and 0.75. For the “strong-sparse” scenario, each causal SNP affects *m* = 10 randomly selected genes and the corresponding values in *B* were simulated from a uniform distribution between 1.5 and 2. The different simulation scenarios are summarized in Table 1.

**TABLE 1:** Simulation scenarios.

Chromosome	#Causal SNPs	Scenario	Method	Screening
Chr 1	60	weak-dense	FastLORS	LORS
	200	strong-sparse	LORSEN	HC
	400		LORS	
Chr 1 + Chr 21	45 + 15	weak-dense	FastLORS	LORS
	150 + 50	strong-sparse	LORSEN	HC
	300 + 100		LORS	

## 3 Results

### 3.1 Simulation Results

The number of selected SNPs and the number of selected causal SNPs from two screening methods under different simulation scenarios are summarized in Table 2. Several conclusions emerge from Table 2. First, when the number of samples is much smaller than the number of SNPs and the number of causal SNPs is larger than the number of samples, HC-Screening is seemingly not an appropriate screening tool. This is because the number of causal SNPs retained after the HC-Screening is much smaller than the actual number of causal SNPs, resulting in possible power loss in subsequent analysis. Second, even when the number of causal SNPs is smaller than the number of samples, from Table 2, we still observed that the LORS-Screening retains more causal SNPs than the HC-Screening. Of course, the HC-Screening reduces much computational burden especially when the number of samples is much smaller than the number of SNPs.

**TABLE 2:** Results of the HC-Screening and the LORS-Screening with ten replicates for each simulation scenario.

Chromosome	#Causal	Scenario	Screening	Average#Slected SNPs	Average#Selected Causal SNPs
	SNPs				
Chr 1	60	weak-dense	LORS	1,017	43
			HC	165	7
		strong-sparse	LORS	1,023	60
			HC	165	9
	200	weak-dense	LORS	1,036	130
			HC	165	20
		strong-sparse	LORS	1,095	199
			HC	165	28
	400	weak-dense	LORS	1,045	237
			HC	165	39
		strong-sparse	LORS	1,142	346
			HC	165	44
Chr 1 + Chr 21	45 + 15	weak-dense	LORS	1,044	46
			HC	165	7
		strong-sparse	LORS	1,065	60
			HC	165	10
	150 + 50	weak-dense	LORS	1,064	136
			HC	165	20
		strong-sparse	LORS	1,123	199
			HC	165	28
	300 + 100	weak-dense	LORS	1,064	244
			HC	165	37
		strong-sparse	LORS	1,188	361
			HC	165	44

The area under the curve (AUC) was used to compare the performance between LORSEN and two existing methods, LORS (Yang et al., 2013) and FastLORS (Jeng et al., 2020). For each scenario, we repeated the simulation ten times. We considered the joint modeling of multiple SNPs and multiple gene expression levels with the SNP screening and without the SNP screening. The results without the SNP screening before the eQTL mapping under different simulation scenarios are presented in Tables 3, 4. From Tables 3, 4, we can see that the average AUC of LORSEN is uniformly larger than those of LORS and FastLORS in the weak-dense scenarios across different number of causal SNPs no matter the SNPs are from single chromosome (Chr 1) or two chromosomes (Chr 1 + Chr 21). For the strong-sparse scenarios, FastLORS achieves the relatively larger AUC than LORS and LORSEN. For a fixed number of causal SNPs, each method achieves the larger AUC value in the stong-sparse scenario than in the weak-dense scenario. For each method under each simulation scenario, the AUCs in Tables 3, 4 are similar, implying that each of three methods has the similar power to detect *cis*-eQTLs and *trans*-eQTLs.

**TABLE 3:** The average AUC and 95% confidence interval without the SNP screening with ten replicates for each simulation scenario. SNPs are only from chromosome 1. For each simulation scenario, the highest AUC is in bold.

		#Causal SNPs		
Scenario	Method	60	200	400
weak-dense	FastLORS	0.514 (0.511, 0.517)	0.582 (0.580, 0.584)	0.581 (0.580, 0.582)
	LORSEN	**0.651** (0.648, 0.654)	**0.649** (0.647, 0.651)	**0.630** (0.629, 0.631)
	LORS	0.502 (0.499, 0.505)	0.514 (0.512, 0.516)	0.515 (0.514, 0.516)
strong-sparse	FastLORS	0.762 (0.755, 0.769)	**0.840** (0.837, 0.843)	**0.810** (0.807, 0.813)
	LORSEN	0.823 (0.817, 0.829)	0.834 (0.831, 0.837)	0.774 (0.771, 0.777)
	LORS	**0.824** (0.818, 0.830)	0.819 (0.815, 0.823)	0.754 (0.751, 0.757)

**TABLE 4:** The average AUC and 95% confidence interval without the SNP screening with ten replicates for each simulation scenario. SNPs are from chromosome 1 and chromosome 21. For each simulation scenario, the highest AUC is in bold.

		#Causal SNPs		
Scenario	Method	60	200	400
weak-dense	FastLORS	0.530 (0.527, 0.533)	0.567 (0.565, 0.569)	0.575 (0.574, 0.576)
	LORSEN	**0.658** (0.655, 0.661)	**0.679** (0.677, 0.681)	**0.625** (0.624, 0.626)
	LORS	0.503 (0.500, 0.506)	0.510 (0.508, 0.512)	0.514 (0.513, 0.515)
strong-sparse	FastLORS	0.774 (0.767, 0.781)	**0.826** (0.822, 0.830)	**0.813** (0.810, 0.816)
	LORSEN	**0.814** (0.807, 0.821)	0.810 (0.806, 0.814)	0.788 (0.785, 0.791)
	LORS	0.813 (0.806, 0.820)	0.801 (0.797, 0.805)	0.756 (0.753, 0.759)

The results with the SNP screening before eQTL mapping under different simulation scenarios are presented in Tables 5, 6. As we have mentioned, the LORS-Screening keeps more SNPs in the analysis, thus retains more causal SNPs than the HC-Screening does. Each method with the LORS-Screening has the larger AUC values than it with the HC-Screening. From Tables 5, 6, we can see that the AUC values of methods with the HC-Screening are quite close to 0.5, which indicates that the HC-Screening can essentially lead to the loss of power of methods. With the LORS-Screening, similar to the non-screening cases, LORSEN has better performance than LORS and FastLORS in the weak-dense scenarios and LORSEN and FastLORS perform similarly and slightly better than LORS in the strong-sparse scenarios. Finally, we find that for the weak-dense scenarios, each method without the SNP screening before joint modeling achieves the larger AUC values than it with the SNP screening. However, for the strong-sparse scenarios, each method with the LORS-Screening before joint modeling achieves the larger AUC values than it without the SNP screening. This may be due to that there are a large number of SNP-gene pairs with the weak association effects in the weak-dense scenarios and many causal SNPs may not be selected by the pre-screening methods. So, in the weak-dense scenarios with the use of pre-screening methods, the computational cost and the detection power can be reduced at the same time. In the strong-sparse scenarios, there are a smaller number of SNP-gene pairs with the stronger association effects than in the weak-dense scenarios, and it is expected that most of the causal SNPs will be selected by the pre-screening methods. Therefore, for the strong-sparse scenarios, the use of pre-screening methods reduce the computational cost while still retain the high detection power.

**TABLE 5:** The average AUC and 95% confidence interval with the SNP screening with ten replicates for each simulation scenario. SNPs are only from chromosome 1. For each simulation scenario, the highest AUC is in bold.

			Screening	
Scenario	#Causal SNPs	Method	HC	LORS
weak-dense	60	FastLORS	0.514 (0.511, 0.517)	0.596 (0.593, 0.599)
		LORSEN	**0.515** (0.512, 0.518)	**0.618** (0.615, 0.621)
		LORS	0.503 (0.500, 0.506)	0.541 (0.538, 0.544)
	200	FastLORS	**0.512** (0.510, 0.514)	0.583 (0.581, 0.585)
		LORSEN	0.511 (0.509, 0.513)	**0.592** (0.590, 0.594)
		LORS	0.502 (0.500, 0.504)	0.519 (0.517, 0.521)
	400	FastLORS	**0.510** (0.509, 0.511)	**0.557** (0.556, 0.558)
		LORSEN	0.509 (0.508, 0.510)	0.547 (0.546, 0.548)
		LORS	0.502 (0.501, 0.503)	0.511 (0.510, 0.512)
strong-sparse	60	FastLORS	**0.565** (0.557, 0.573)	0.900 (0.895, 0.905)
		LORSEN	0.558 (0.550, 0.566)	**0.903** (0.898, 0.908)
		LORS	0.560 (0.552, 0.568)	0.897 (0.892, 0.902)
	200	FastLORS	**0.552** (0.548, 0.556)	**0.894** (0.891, 0.897)
		LORSEN	0.544 (0.540, 0.548)	**0.894** (0.891, 0.897)
		LORS	0.543 (0.539, 0.547)	0.874 (0.871, 0.877)
	400	FastLORS	**0.536** (0.533, 0.539)	**0.797** (0.794, 0.800)
		LORSEN	0.523 (0.520, 0.526)	0.782 (0.779, 0.785)
		LORS	0.528 (0.525, 0.531)	0.738 (0.735, 0.741)

**TABLE 6:** The average AUC and 95% confidence interval with the SNP screening with ten replicates for each simulation scenario. SNPs are from chromosome 1 and chromosome 21. For each simulation scenario, the highest AUC is in bold.

			Screening	
Scenario	#Causal SNPs	Method	HC	LORS
weak-dense	60	FastLORS	0.518 (0.515, 0.521)	0.606 (0.603, 0.609)
		LORSEN	**0.518** (0.515, 0.521)	**0.629** (0.626, 0.632)
		LORS	0.505 (0.502, 0.508)	0.544 (0.541, 0.547)
	200	FastLORS	**0.512** (0.510, 0.514)	0.591 (0.589, 0.593)
		LORSEN	**0.512** (0.510, 0.514)	**0.615** (0.613, 0.617)
		LORS	0.503 (0.501, 0.505)	0.524 (0.522, 0.526)
	400	FastLORS	**0.510** (0.509, 0.511)	**0.563** (0.562, 0.564)
		LORSEN	0.507 (0.506, 0.508)	0.556 (0.555, 0.557)
		LORS	0.501 (0.500, 0.502)	0.511 (0.510, 0.512)
strong-sparse	60	FastLORS	**0.570** (0.562, 0.578)	0.891 (0.886, 0.896)
		LORSEN	0.563 (0.555, 0.571)	**0.906** (0.901, 0.911)
		LORS	0.564 (0.556, 0.572)	0.891 (0.886, 0.896)
	200	FastLORS	**0.553** (0.549, 0.557)	**0.904** (0.901, 0.907)
		LORSEN	0.547 (0.543, 0.551)	**0.904** (0.901, 0.907)
		LORS	0.544 (0.540, 0.548)	0.883 (0.880, 0.886)
	400	FastLORS	**0.534** (0.531, 0.537)	**0.821** (0.818, 0.824)
		LORSEN	0.524 (0.521, 0.527)	0.813 (0.810, 0.816)
		LORS	0.525 (0.522, 0.528)	0.765 (0.762, 0.768)

Our simulation results showed that LORSEN is more powerful to identify weak signals, while it does not have obvious advantage in identifying strong signals compared to LORS. Therefore, we performed additional simulation studies in which the causal variants have mixed weak and strong effects. Specifically, the half of the causal variants had the weak effects and their effects were generated from a uniform distribution between 0.25 and 0.75, while the other half of the causal variants had the strong effects and their effects were generated from a uniform distribution between 1.5 and 2. The number of causal SNPs was set as 60, 200, or 400. The number of genes affected by one causal SNP was set as 50. The AUCs and corresponding 95% confidence intervals are presented in Supplementary Table S1. From the results in Supplementary Table S1, we can see that LORSEN has the overall highest detection power when the number of causal SNPs is large. It is well known that the rare variants play an important role in the etiology of human complex diseases. Therefore, it is necessary to assess the performance of eQTL mapping methods when most of causal variants are rare. We conducted simulations in which the proportion of rare causal variants was set to be 50 and 75%. Here, the variants with minor allel frequency (MAF) less than 0.03 were considered as the rare variants. The number of causal variants was set as 200. The results from different simulation scenarios (weak-dense and strong-sparse) are presented in Supplementary Table S2. From Supplementary Table S2, we can see that when the proportion of causal rare variants is 50%, the AUCs of FastLORS are slightly higher than the AUCs of LORSEN. However, when the proportion of causal rare variants is 75%, the AUCs of LORSEN are at least 10% higher AUCs than the AUCs of FastLORS and about 20% higher than the AUCs of LORS. Our results show that LORSEN has the higher power in detecting rare causal variants. To see how the detection power of LORSEN is affected by the positive and negative effects, we conducted simulations in which the half of the causal variants had the positive effects on genes and the other half of the causal variants had the negative effects on genes. The results from different simulation scenarios (weak-dense and strong-sparse with 60, 200, and 400 causal variants) are presented in Supplementary Table S3. From Supplementary Table S3, we can see that LORSEN achieves the highest AUCs in almost all simulation scenarios, which implies that the detection power of LORSEN is not affected by the effect directions of causal variants.

In addition to AUC, a commonly used measure to assess the performance of methods for eQTL mapping, we also reported the false positive rates (FPRs) based on four thresholds for the regression coefficients: 0, 10^–12^, 10^–6^, 10^–4^. From the Supplementary Figures S1–S3, we can see that FastLORS has the highest FPRs in almost all scenarios, and the FPRs of FastLORS are quite sensitive to the thresholds: the FPRs of FastLORS decrease dramatically for large thresholds. LORS has the smallest FPRs in all simulation scenarios. For LORSEN, it has the small and comparable FPRs with LORS when the effects of the causal variants are all weak or are a mixture of weak and strong effects. LORSEN has the large FPRs when the effects of the causal variants are all strong.

A number of conclusions emerge from the results based on our extensive simulation studies. First, the HC-Screening method retains much smaller number of SNPs than the LORS-Screening method. Second, when all the SNPs are not filtered with the SNP screening method and are used in the analysis, LORSEN is the most powerful method to identify weak signals, while it does not have obvious advantage in identifying strong signals compared to LORS and FastLORS. LORSEN still performs the best with the mixture of the strong and weak effects when the number of causal variants is large. Third, when the SNPs are first filtered with the HC-Screening method, FastLORS performs the best in all simulation scenarios. With the LORS-Screening method, LORSEN has the highest detection power in most of simulation scenarios. Fourth, LORSEN outperforms FastLORS and LORS when a large portion of the causal SNPs are rare and when the causal variants have a mixture of positive and negative effects.

### 3.2 Real Data Analysis Results

**FIGURE 1:**
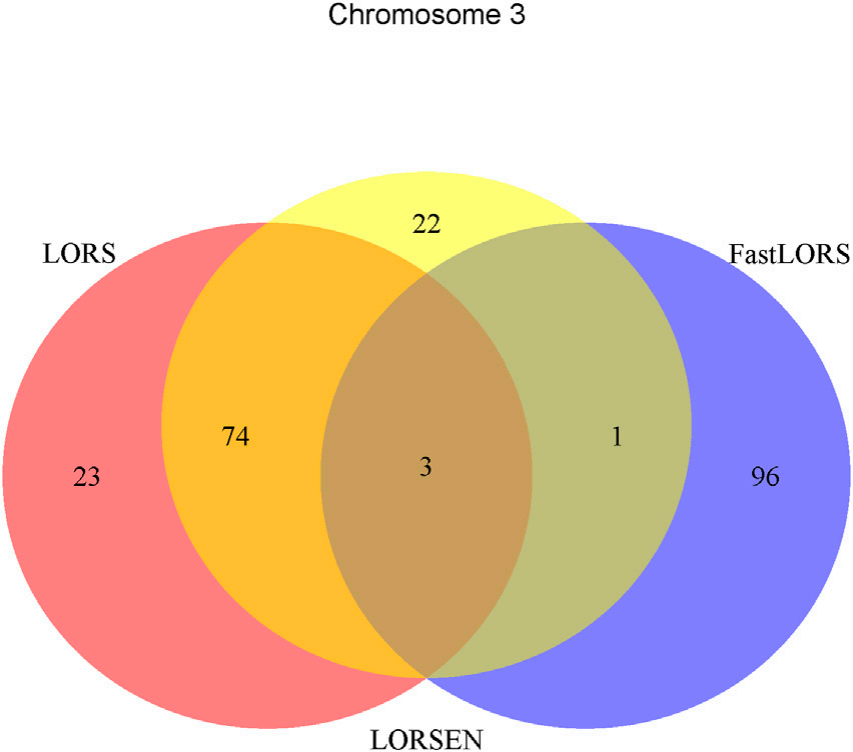
The top 100 SNP-probe pairs identified by FastLORS, LORSEN, and LORS on Chromosome 3.

**FIGURE 2:**
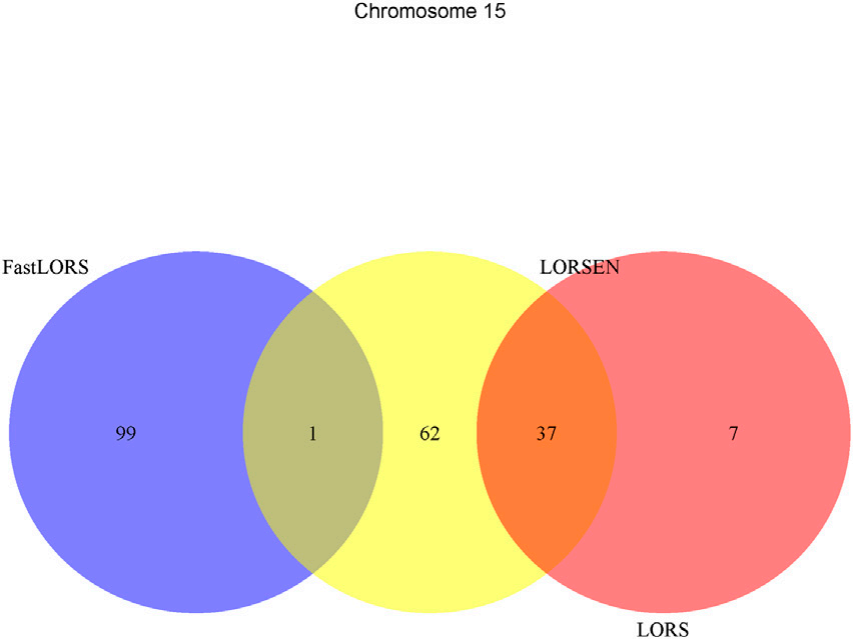
The top 100 SNP-probe pairs identified by FastLORS, LORSEN, and LORS on Chromosome 15.

**FIGURE 3:**
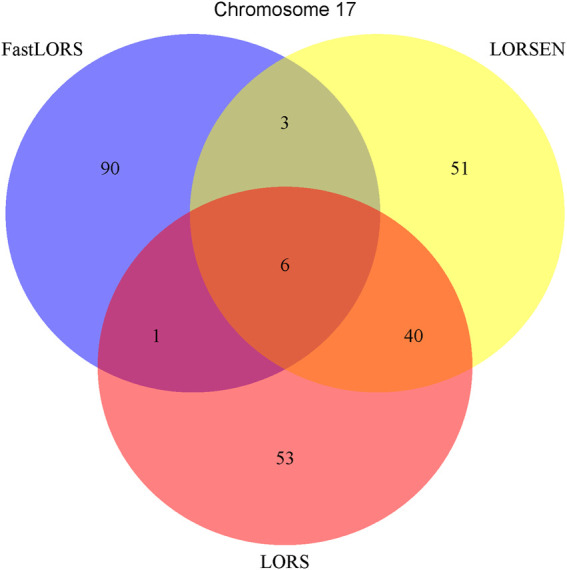
The top 100 SNP-probe pairs identified by FastLORS, LORSEN, and LORS on Chromosome 17.

**FIGURE 4:**
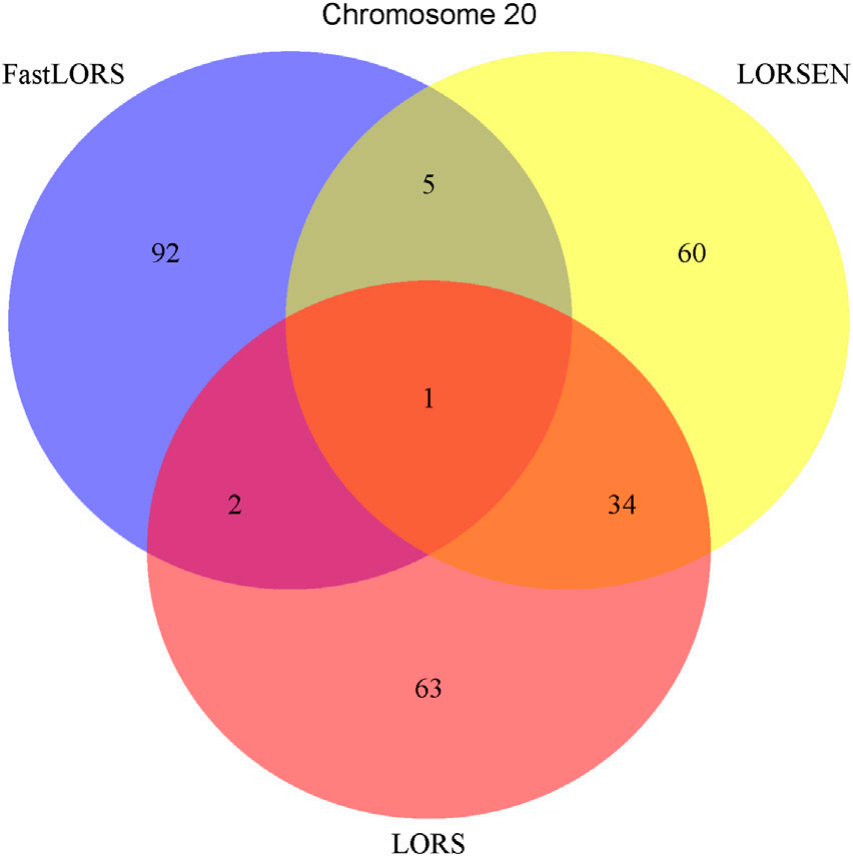
The top 100 SNP-probe pairs identified by FastLORS, LORSEN, and LORS on Chromosome 20.

To illustrate our method in real data analysis, we also applied LORS-LORSEN (LORSEN with the LORS-Screening), LORS-LORS (LORS with the LORS-Screening) and HC-FastLORS (FastLORS with the HC-Screening) to the HapMap3 data. Here, we focused on Asian samples (CHB and JPT) in the HapMap3 data and selected four chromosomes for the analysis. SNP genotype data and gene expression data are publicly available, and can be downloaded from ftp://ftp.ncbi.nlm.nih.gov/hapmap/genotypes/hapmap3_r3/plink_format/and http://www.ebi.ac.uk/arrayexpress/experiments/E-MTAB-264/, respectively. Because the set of samples with the SNP genotype data and the set of samples with the gene expression data are slightly different, we only kept the samples that have both the SNP genotype data and the gene expression data in the analysis. We removed SNPs with missing values, and performed the LD pruning using PLINK with its default parameters (window size: 50; moving window increment: five SNPs; cutoff value of *R* ^2^: 0.5). After the data pre-processing, a total of 160 samples (CHB: 79; JPT: 81) were included in analysis. The number of SNPs and the number of genes with the expression used in the analysis on chromosome 3 are 4,086 and 1,075, on chromosome 15 are 2,235 and 612, on chromosome 17 are 2,226 and 1,098, on chromosome 20 are 1,863 and 606, respectively. Since the significance tests generally cannot be performed for the penalization based regression models, we focused on the top 100 SNP-probe pairs with the largest absolute regression coefficients. From the Venn diagrams (Figures 1–4), we notice that there is a large overlap between the eQTLs identified by LORS-LORS and LORS-LORSEN. However, there is a small overlap between the eQTLs identified by HC-FastLORS and LORS-LORS (or LORS-LORSEN). For example, among the top 100 SNP-probe pairs identified on Chromosome 3 (Figure 1), LORS-LORS and LORS-LORSEN share 77 SNP-probe pairs in common, while LORS-LORSEN and HC-FastLORS only share four SNP-probe pairs in common and LORS-LORS and HC-FastLORS share three SNP-probe pairs in common. This observation is consistent with the observation from (Jeng et al., 2020) which also noticed that there is a small overlap between the SNP-probe pairs identified by LORS-LORS and HC-FastLORS. Additionally, as adopted in (Jeng et al., 2020), we classified the detected eQTL as local if the physical distance between the SNP and the probe midpoint is less than 250 kb or as distant if the distance is greater than 5 mb following the criterion described in (Westra et al., 2013). For each chromosome, we report our findings on the top ten identified SNP-probe pairs in Table 7 and Supplementary Tables S4–S6 (see Supplementary Material). From Table 7, we can see that the SNPs in the top ten SNP-probe pairs identified by HC-FastLORS are all *trans*-eQTLs. As a comparison, seven SNPs in the top ten SNP-probe pairs identified by LORS-LORSEN are *cis*-eQTLs and two SNPs are *trans*-eQTLs. Five SNPs in the top ten SNP-probe pairs identified by LORS-LORS are *cis*-eQTLs and four SNPs are *trans*-eQTLs. LORS-LORSEN and LORS-LORS share seven SNP-probe pairs while LORS-LORSEN and LORS-LORS do not share any SNP-probe pair with HC-FastLORS. In addition, the coefficients obtained from HC-FastLORS are ten-fold smaller than those obtained from LORS-LORSEN and LORS-LORS. This indicates that the findings of LORS-LORSEN and LORS-LORS may be more convincing.

**Algorithm 1:** FISTA with constant step size.

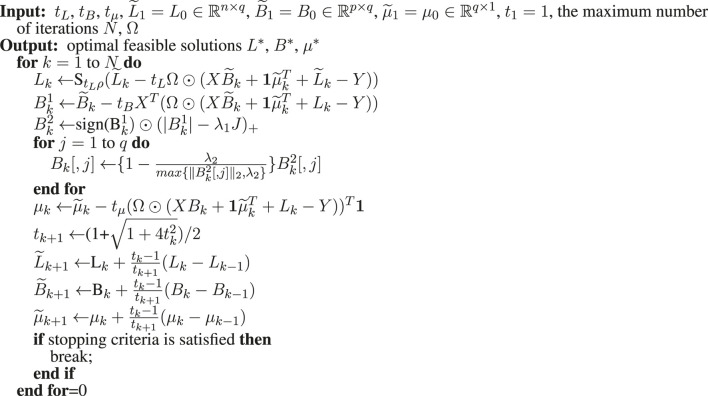

**TABLE 7:** Top ten detected SNP-probe pairs for chromosome 3. The SNP-probe pairs that are confirmed in seeQTL database are in bold.

Method	SNP	Probe (gene)	Association coefficient	Distance	Class
HC-FastLORS	rs13084976	ILMN_1657373 (LEPREL1)	0.0430	188.72 mb	distant
	rs17029694	ILMN_1657373 (LEPREL1)	0.0424	188.49 mb	distant
	rs12494696	ILMN_1812093 (UTS2D)	0.0322	189.72 mb	distant
	rs2322212	ILMN_1756501 (ST6GAL1)	0.0310	184.74 mb	distant
	rs17029694	ILMN_1708743 (NT5DC2)	0.0303	49.86 mb	distant
	rs2322212	ILMN_1686920 (CCDC58)	0.0300	120.03 mb	distant
	rs7647780	ILMN_1762084 (DNASE1L3)	0.0292	57.51 mb	distant
	rs1516347	ILMN_1726020 (LOC652670)	0.0278	75.49 mb	distant
	rs13061928	ILMN_1692261 (EPHB1)	0.0273	133.55 mb	distant
	rs1377213	ILMN_1698934 (CMTM7)	0.0270	26.76 mb	distant
LORS-LORSEN	rs1505587	ILMN_1657373 (LEPREL1)	0.3336	127.69 mb	distant
	rs6807033	ILMN_1787750 (CD200)	0.2796	4.163 kb	local
	rs11914577	ILMN_1700967 (C3orf59)	0.2245	113.51 kb	local
	rs1403719	ILMN_1771599 (PLOD2)	0.1963	25.06 mb	distant
	rs628267	ILMN_1760509 (EOMES)	0.1941	302.30 kb	
	**rs4016435**	**ILMN_1757350 (CTNNB1)**	**0.1908**	**27.772 kb**	**local**
	**rs16839507**	**ILMN_1761058 (ACAD11)**	**0.1856**	**117.942 kb**	**local**
	**rs693430**	**ILMN_1657708 (MGLL)**	**0.1796**	**86.074 kb**	**local**
	**rs693430**	**ILMN_1707310 (MGLL)**	**0.1710**	**47.617 kb**	**local**
	rs1498090	ILMN_1793724 (C3orf31)	0.1662	58.605 kb	local
LORS-LORS	rs1505587	ILMN_1657373 (LEPREL1)	1.2549	127.69 mb	distant
	rs6807033	ILMN_1787750 (CD200)	0.5621	4.163 kb	local
	rs4857653	ILMN_1700967 (C3orf59)	0.3640	16.16 mb	distant
	rs11914577	ILMN_1700967 (C3orf59)	0.2984	113.514 kb	local
	rs1403719	ILMN_1771599 (PLOD2)	0.2824	25.06 mb	distant
	rs628267	ILMN_1760509 (EOMES)	0.2439	302.302 kb	
	**rs4016435**	**ILMN_1757350 (CTNNB1)**	**0.2404**	**27.772 kb**	**local**
	**rs16839507**	**ILMN_1761058 (ACAD11)**	**0.2338**	**117.942 kb**	**local**
	rs3773014	ILMN_1762084 (DNASE1L3)	0.2268	29.187 kb	local
	rs1799977	ILMN_1688392 (ZBED2)	0.2234	75.77 mb	distant

To further validate our findings, we searched an existing database called seeQTL (Xia et al., 2011). seeQTL (https://seeqtl.org/) records the eQTLs identified from a meta-analysis (consensus eQTLs) from the HapMap human lymphoblastoid cell lines. A total of fourteen SNP-probe pairs were found in seeQTL and were listed in Table 8. Among them, two SNP-probe pairs were identified by HC-FastLORS only, three were identified by LORS-LORSEN only, two were identified by LORS-LORS only, seven were identified by both LORS-LORSEN and LORS-LORS, and one was identified by all three methods. To further validate these fourteen SNP-probe pairs, we searched the eQTL web-browser (http://www.gtexportal.org/home/) built by the Genotype-Tissue Expression Project (GTEx) (https://www.genome.gov/Funded-Programs-Projects/Genotype-Tissue-Expression-Project) to see if those SNP-probe (gene) pairs are listed as the eQTLs and/or sQTLs (splicing quantitative trait locus). A total of seven SNP-probe pairs were also found in GTEx and were presented in Table 8. Among seven SNP-probe pairs found both in seeQTL and GTEx, one SNP-probe pair was identified by all three methods, five SNP-probe pairs were identified by both LORS-LORSEN and LORS-LORS, and one SNP-probe pair was identified by LORS-LORSEN only.

**TABLE 8:** The SNP-probe pairs found in seeQTL database out of the top ten SNP-probe pairs for chromosomes 3, 15, 17, and 20, respectively.

Chromosome	SNP	Probe (gene)	Method	Information from GTEx
3	rs4016435	ILMN_1757350 (CTNNB1)	LORS-LORSEN, LORS-LORS	Not found in GTEx
3	rs16839507	ILMN_1761058 (ACAD11)	LORS-LORSEN, LORS-LORS	Multiple hits for eQTLs and sQTLs
3	rs693430	ILMN_1657708 (MGLL)	LORS-LORSEN	Not found in GTEx
3	rs693430	ILMN_1657708 (MGLL)	LORS-LORSEN	Not found in GTEx
15	rs7162538	ILMN_1784364 (STARD5)	LORS-LORSEN, LORS-LORS	Multiple hits for eQTLs and sQTLs
15	rs1347069	ILMN_1795822 (DIS3L)	LORS-LORSEN, LORS-LORS	Multiple hits for eQTLs and sQTLs
15	rs2292114	ILMN_1795524 (C15orf44)	LORS-LORS	Not found in GTEx
17	rs4968140	ILMN_1706959 (TIMM22)	HC-FastLORS	Not found in GTEx
17	rs4251704	ILMN_1773352 (CCL5)	LORS-LORSEN	A single hit for sQTLs
17	rs17657522	ILMN_1697227 (USP36)	LORS-LORSEN, LORS-LORS	Multiple hits for eQTLs and sQTLs
17	rs4968140	ILMN_1706959 (TIMM22)	LORS-LORSEN, LORS-LORS	Not found in GTEx
17	rs9905601	ILMN_1750511 (NT5C3L)	LORS-LORS	Not found in GTEx
20	rs16989514	ILMN_1721128 (TOMM34)	LORS-LORSEN, LORS-LORS	Multiple hits for eQTLs and sQTLs
20	rs6041750	ILMN_1702237 (FKBP1A)	HC-FastLORS, LORS-LORSEN, LORS-LORS	Multiple hits for eQTLs and sQTLs

A number of conclusions emerge from the results based on HapMap3 data. First, there is a large overlap between the SNP-probe pairs identified by LORS-LORS and LORS-LORSEN but there is a small overlap between the SNP-probe pairs identified by HC-FastLORS and LORS-LORS (or LORS-LORSEN). Second, LORS-LORS and LORS-LORSEN perform similarly and have higher detection power than HC-FastLORS since LORS-LORS and LORS-LORSEN have identified more SNP-probe pairs that are also found in seeQTL and GTEx. Third, five out of seven SNP-probe pairs identified by both LORS-LORS and LORS-LORSEN and found in seeQTL are also found in GTEx, thus it may be beneficial to combine the results from multiple methods to generate a list of SNP-probe pairs for further investigation.

## 4 Discussion

As more human gene expression data become available, fast and efficient statistical and computational methods are needed to fully take advantage of such data to investigate the relationship between genetic variants and gene expression levels to further reveal the genetic mechanisms that underlie human complex diseases. However, most existing methods are built on small-scale samples and not applicable to human-size datasets. In this paper, we proposed a new low rank penalized regression method (LORSEN) to detect eQTLs. We developed a fast and efficient algorithm to solve optimization problems arising from our methods based on proximal gradient methods. Comprehensive simulation studies showed that LORSEN outperformed two commonly used methods, LORS and FastLORS, in many simulation scenarios. From our simulation results, we can briefly conclude that, first, LORSEN is more powerful in detecting eQTLs which are rare and/or have weak effects. This is especially an appealing advantage since it is expected that a portion of causal variants are rare and/or have the weak effects in the real world. Second, LORSEN is more powerful when some causal variants have the positive effects and the other causal variants have the negative effects.

Since there are a large number of SNPs and genes to be included in the eQTL mapping and it is expected that only a small portion of SNPs will affect the gene expression levels, a number of pre-screening methods have been developed. In this paper, we used the LORS-Screening (Yang et al., 2013) and the HC-Screening (Jeng et al., 2020). We found that the HC-Screening retained much smaller number of SNPs than the LORS-Screening. Both the LORS-Screening and the HC-Screening can reduce the computational cost, but they may also reduce the detection power in the eQTL mapping, depending on the association patterns between SNPs and gene expression levels. Since we do not know such association patterns in real studies, we should be cautious to apply such pre-screening methods.

There are several limitations for LORSEN. First, as a method based on the penalized regression model, we can rank the SNP-gene pairs in terms of the regression coefficients obtained from LORSEN, but cannot perform the significance test. Second, the computational time of LORSEN depends on many factors such as the number of candidate values of hyperparameters, the initial values of hyperparameters, and the number of samples. The computation was performed parallelly using software R (verson 4.1.1) and 16 cores on a server with 64 Intel(R) Xeon(R) Gold 6130 CPUs @ 2.10 GHz. From Supplementary Table S7, we can see that, as expected, LORSEN costs much more time in parameter tuning than other two methods due to the exhaustive grid search. The grid search is easy to be implemented but is computationally intensive. It may not be feasible for large scale data. A more efficient strategy is desirable.

It has shown that the incorporation of the SNP correlation and the gene interaction network can potentially increase the power of detecting eQTLs (Kim and Xing, 2009; Chen et al., 2012; Kim and Xing, 2012; Cheng et al., 2014). We expect that our method can be improved if we use the prior knowledge of correlation structures of SNPs and genes to refine the penalty terms in optimization problems.

## Data Availability

The original contributions presented in the study are included in the article/Supplementary Material, further inquiries can be directed to the corresponding author.

## Appendix A

Lemma 1: For each *τ* ⩾0 and $Y\in R^{n_{1}\times n_{2}}$ , the solution of

$$\underset{X}{min}\;\frac{1}{2}\|X-Y{\|}_{F}^{2}+\tau \|X{\|}_{*}$$
(16)

is $S_{\tau }\left(Y\right)≔US_{\tau }\left(\Sigma \right)V^{T}\left(=Prox_{\tau \|\cdot {\|}_{*}}\left(Y\right)\right)$ , where $S_{\tau }\left(\Sigma \right)=diag\left(\left\{{\left(\sigma_{i}-\tau \right)}_{+}\right\}\right)$ , *Y* = *U*Σ*V* ^*T*^, the singular value decomposition of matrix *Y*, $\Sigma =diag\left({\left\{\sigma_{i}\right\}}_{1⩽i⩽r}\right)$ , *r* is the rank of *Y*. *S*_*τ*_(⋅) is called singular value shrinkage operator.

Proof: see (Cai et al., 2010) or (Mazumder et al., 2010).

Lemma 2: For each fixed non-negative *λ* and $v\in R^{n}$ , the solution of

$$\underset{x}{min}\;\frac{1}{2}\|x-v{\|}_{2}^{2}+\frac{\lambda }{2}\|x{\|}_{2}^{2}$$
(17)

is ${\left(Prox_{\frac{\lambda }{2}\|\cdot {\|}_{2}^{2}}\left(v\right)\right)}_{i}=sign\left(v_{i}\right){\left(|v_{i}|-\lambda \right)}_{+}$ , *i* = 1, 2, …, *n*, known as the (elementwise) soft thresholding operator.

Proof: see (Parikh and Boyd, 2014).

Lemma 3: For each fixed non-negative *ρ* and $v\in R^{n}$ , the solution of

$$\underset{x}{min}\;\frac{1}{2}\|x-v{\|}_{2}^{2}+\rho \|x{\|}_{1}$$
(18)

is $Prox_{\rho \|\cdot {\|}_{1}}\left(v\right)=\left(1-\frac{\rho }{max\left\{\|v{\|}_{2},\rho \right\}}\right)v$ .

Proof: see (Parikh and Boyd, 2014).

Lemma 4: (soft-impute algorithm) For the optimization problem

$$\begin{array}{ll} & \underset{X}{min}\;\frac{1}{2}\|P_{\Omega }\left(Y-X\right){\|}_{F}^{2}+\tau \|X{\|}_{*}\\ & =\underset{X}{min}\;\frac{1}{2}\|\left[P_{\Omega }\left(Y\right)+P_{\Omega^{⊥}}\left(X\right)\right]-X{\|}_{F}^{2}+\tau \|X{\|}_{*}, \end{array}$$

the optimization solution can be obtained via updating *X* using $X\leftarrow S_{\tau }\left(P_{\Omega }\left(Y\right)+P_{\Omega^{⊥}}\left(X\right)\right)$ with an arbitrary initialization.

Proof: see (Mazumder et al., 2010).

Theorem 1: A sufficient condition for *Prox*_*f*+*g*_ = *Prox*_*f*_◦*Prox*_*g*_ is ∀x∈H , *∂g*(*Prox*_*f*_(*x*)) ⊇ *∂g*(*x*), where H represents Hilbert space and ◦ represents composition of two operators.

Proof: see (Yu, 2013). Details of Confidence Interval of AUC We followed the method used in (Hanley and McNeil, 1982) to calculate the 95% confidence interval (CI) of AUC. Let AUC^ and Var(AUC^) denote the sample mean and the estimated variance of AUCs from ten replicates, respectively, the 95% CI of average AUC was calculated using the following formula:

AUC^¯±1.96Var(AUC^)/10.
(19)

We used the following formula (Hanley and McNeil, 1982) to calculate Var(AUC^) :

Var(AUC^)=q0+(n1−1)q1+(n2−1)q2n1n2,
(20)

where q0=AUC^(1−AUC^) , q1=AUC^2−AUC^−AUC^2 , q2=2AUC^21+AUC^−AUC^2 , *n*_1_ is the number of true positives, and *n*_2_ is the number of true negatives.
