# Supplementary material for: LORSEN: Fast and Efficient eQTL Mapping With Low Rank Penalized Regression
Source: Front Genet. 2021 Nov 17;12:690926. doi: 10.3389/fgene.2021.690926 (PMC8636089; doi:10.3389/fgene.2021.690926)
Supplement: Supplementary file 1 [file Presentation1.zip › FigureS2.pdf]

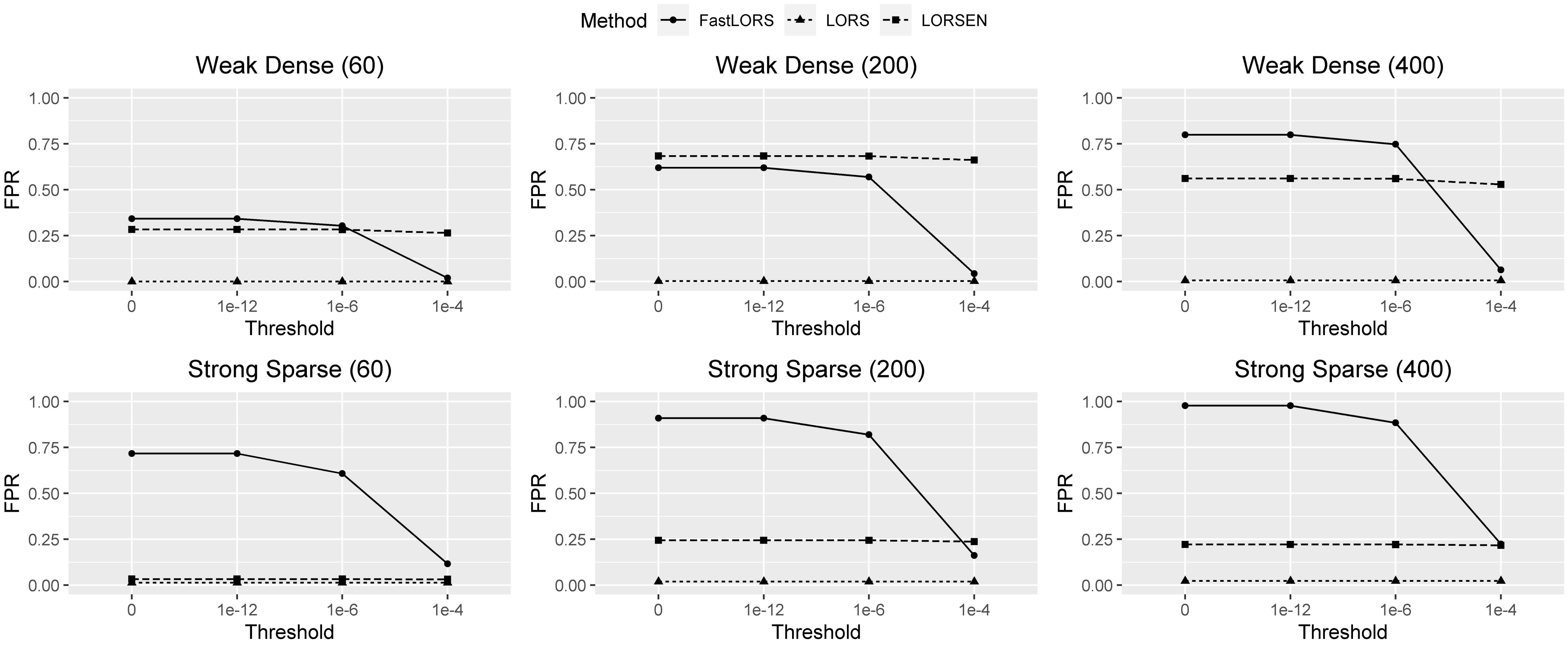

Method
FastLORS
LORS
LORSEN
Weak Dense (60)
Weak Dense (200)
Weak Dense (400)
Strong Sparse (60)
Strong Sparse (200)
Strong Sparse (400)
FPR
0.00
0.25
0.50
0.75
1.00
Threshold
0
1e-12
1e-6
1e-4
