# Supplementary material for: LORSEN: Fast and Efficient eQTL Mapping With Low Rank Penalized Regression
Source: Front Genet. 2021 Nov 17;12:690926. doi: 10.3389/fgene.2021.690926 (PMC8636089; doi:10.3389/fgene.2021.690926)
Supplement: Supplementary file 2 [file Presentation2.pdf]

Supplementary Material

1 SUPPLEMENTARY TABLES

Table S1. The average AUC and 95% confidence interval with mixed strong and weak signals without the SNP screening with ten replicates for each simulation scenario. SNPs are only from chromosome 1. For each simulation scenario, the highest AUC is in bold.

Methods	#Causal SNPs		
	60	200	400
FastLORS	**0.694 (0.691 , 0.697)**	0.659 (0.657 , 0.661)	0.625 (0.624 , 0.626)
LORSEN	0.684 (0.681 , 0.687)	**0.664 (0.662 , 0.666)**	**0.641 (0.640 , 0.642)**
LORS	0.622 (0.619 , 0.625)	0.588 (0.586 , 0.590)	0.557 (0.556 , 0.558)

Table S2. The average AUC and 95% confidence interval without the SNP screening with ten replicates for each simulation scenario with two different proportions of causal rare variants. The total number of causal SNPs is 200. SNPs are only from chromosome 1. For each simulation scenario, the highest AUC is in bold.

Scenario	Method	Proportion of causal rare variants	
		50%	75%
weak-dense	FastLORS	**0.538 (0.536 , 0.540)**	0.516 (0.514 , 0.518)
	LORSEN	0.500 (0.498 , 0.502)	**0.598 (0.596 , 0.600)**
	LORS	0.502 (0.500 , 0.504)	0.502 (0.500 , 0.504)
strong-sparse	FastLORS	**0.662 (0.658 , 0.666)**	0.591 (0.587 , 0.595)
	LORSEN	0.650 (0.646 , 0.654)	**0.658 (0.654 , 0.662)**
	LORS	0.598 (0.594 , 0.602)	0.541 (0.537 , 0.545)

Table S3. The average AUC and 95% confidence interval without the SNP screening with ten replicates for each simulation scenario. Half causal effects are positive and the other half causal effects are negative. SNPs are only from chromosome 1. For each simulation scenario, the highest AUC is in bold.

Scenario	Method	#Causal SNPs		
		60	200	400
weak-dense	FastLORS	0.509 (0.506 , 0.512)	0.512 (0.510 , 0.514)	0.512 (0.511 , 0.513)
	LORSEN	**0.604 (0.601 , 0.607)**	**0.604 (0.602 , 0.606)**	**0.582 (0.581 , 0.583)**
	LORS	0.514 (0.511 , 0.517)	0.521 (0.519 , 0.523)	0.529 (0.528 , 0.530)
strong-sparse	FastLORS	0.590 (0.582 , 0.598)	0.659 (0.655 , 0.663)	0.612 (0.609 , 0.615)
	LORSEN	**0.841 (0.835 , 0.847)**	0.774 (0.770 , 0.778)	**0.771 (0.768 , 0.774)**
	LORS	0.838 (0.832 , 0.844)	**0.805 (0.801 , 0.809)**	0.758 (0.755 , 0.761)

Table S4. Top ten detected SNP-probe pairs for chromosome 15. The SNP-probe pairs that are confirmed in seeQTL database are in bold.

Method	SNP	Probe (Gene)	Class
HC-FastLORS	rs12594727	ILMN_1652797 (LOC400451)	distant
	rs17734920	ILMN_1652797 (LOC400451)	distant
	rs12594727	ILMN_1804277 (SPRED1)	distant
	rs4567674	ILMN_1692517 (LOC653381)	distant
	rs12440268	ILMN_1692517 (LOC653381)	distant
	rs1977035	ILMN_1710216 (AVEN)	distant
	rs11633486	ILMN_1690695 (PEX11A)	distant
	rs6606804	ILMN_1665859 (RAB27A)	distant
	rs1977035	ILMN_1693650 (FES)	distant
	rs11634559	ILMN_1748374 (LOC400304)	
LORS-LORSEN	rs6151443	ILMN_1712082 (GCNT3)	local
	rs12441559	ILMN_1712082 (GCNT3)	distant
	rs9635390	ILMN_1656899 (CIB1)	local
	rs16970801	ILMN_1749096 (BCL2L10)	distant
	rs8024414	ILMN_1813430 (TRIM69)	distant
	rs288406	ILMN_1808238 (RBPMS2)	distant
	**rs7162538**	**ILMN_1784364 (STARD5)**	**local**
	rs16957709	ILMN_1792173 (76P)	local
	**rs1347069**	**ILMN_1795822 (DIS3L)**	**local**
	rs3825946	ILMN_1667199 (SQRDL)	local
LORS-LORS	rs6151443	ILMN_1712082 (GCNT3)	local
	rs9635390	ILMN_1656899 (CIB1)	local
	**rs7162538**	**ILMN_1784364 (STARD5)**	**local**
	rs16957709	ILMN_1792173 (76P)	local
	rs12440502	ILMN_1805410 (C15orf48)	distant
	**rs2292114**	**ILMN_1795524 (C15orf44)**	**local**
	**rs1347069**	**ILMN_1795822 (DIS3L)**	**local**
	rs25431	ILMN_1748374 (LOC400304)	distant
	rs3825946	ILMN_1667199 (SQRDL)	local
	rs7177893	ILMN_1689274 (NIPA1)	local

Table S5. Top ten detected SNP-probe pairs for chromosome 17. The SNP-probe pairs that are confirmed in seeQTL database are in bold.

Method	SNP	Probe (Gene)	Class
HC-FastLORS	rs8082184	ILMN_1747419 (PCGF2)	distant
	rs4790694	ILMN_1773352 (CCL5)	distant
	rs3213714	ILMN_1769550 (SLFN5)	distant
	rs2317668	ILMN_1769550 (SLFN5)	distant
	rs12950579	ILMN_1769550 (SLFN5)	distant
	rs17822338	ILMN_1769550 (SLFN5)	distant
	rs4985676	ILMN_1733811 (JUP)	distant
	**rs4968140**	**ILMN_1706959 (TIMM22)**	**local**
	rs6806	ILMN_1810486 (RAB34)	distant
	rs9915773	ILMN_1707448 (CRKRS)	distant
LORS-LORSEN	rs4794776	ILMN_1808301 (MRPL45)	local
	**rs4251704**	**ILMN_1773352 (CCL5)**	**local**
	rs4789267	ILMN_1782778 (FAM100B)	local
	rs3809767	ILMN_1687247 (SPATA20)	local
	**rs17657522**	**ILMN_1697227 (USP36)**	**local**
	rs4796817	ILMN_1697227 (USP36)	local
	rs12952713	ILMN_1750511 (NT5C3L)	distant
	**rs4968140**	**ILMN_1706959 (TIMM22)**	**local**
	rs6504230	ILMN_1747347 (C17orf60)	local
	rs33926631	ILMN_1738027 (BRCA1)	local
LORS-LORS	rs4794776	ILMN_1808301 (MRPL45)	local
	rs3809767	ILMN_1687247 (SPATA20)	local
	**rs17657522**	**ILMN_1697227 (USP36)**	**local**
	rs4796817	ILMN_1697227 (USP36)	local
	**rs9905601**	**ILMN_1750511 (NT5C3L)**	**local**
	rs11868362	ILMN_1733811 (JUP)	distant
	rs4791136	ILMN_1733811 (JUP)	distant
	**rs4968140**	**ILMN_1706959 (TIMM22)**	**local**
	rs6504230	ILMN_1747347 (C17orf60)	local
	rs33926631	ILMN_1738027 (BRCA1)	local

Table S6. Top ten detected SNP-probe pairs for chromosome 20. The SNP-probe pairs that are confirmed in seeQTL database are in bold.

Method	SNP	Probe (Gene)	Class
HC-FastLORS	rs692862	ILMN_1713561 (C20orf103)	distant
	rs530652	ILMN_1814247 (TCFL5)	distant
	rs6084912	ILMN_1791771 (HCK)	distant
	rs16991099	ILMN_1758146 (SIRPA)	
	rs6084217	ILMN_1804822 (SRXN1)	
	rs16991131	ILMN_1666269 (CTSZ)	distant
	**rs6041750**	**ILMN_1702237 (FKBP1A)**	**local**
	rs692862	ILMN_1712347 (SFRS6)	distant
	rs6052369	ILMN_1712347 (SFRS6)	distant
	rs1292244	ILMN_1670841 (CPNE1)	distant
LORS-LORSEN	rs760087	ILMN_1814247 (TCFL5)	local
	rs6115906	ILMN_1751330 (RBCK1)	local
	rs4911408	ILMN_1798014 (EIF2S2)	local
	**rs16989514**	**ILMN_1721128 (TOMM34)**	**local**
	rs2223246	ILMN_1666181 (SDC4)	
	**rs6041750**	**ILMN_1702237 (FKBP1A)**	**local**
	rs1410936	ILMN_1712347 (SFRS6)	distant
	rs2223246	ILMN_1712347 (SFRS6)	local
	rs6103330	ILMN_1712347 (SFRS6)	local
	rs13040414	ILMN_1712347 (SFRS6)	
LORS-LORS	rs6109758	ILMN_1713561 (C20orf103)	
	rs6112999	ILMN_1713561 (C20orf103)	distant
	rs6075584	ILMN_1814247 (TCFL5)	distant
	rs760087	ILMN_1814247 (TCFL5)	local
	**rs16989514**	**ILMN_1721128 (TOMM34)**	**local**
	**rs6041750**	**ILMN_1702237 (FKBP1A)**	**local**
	rs209901	ILMN_1811315 (EEF1A2)	distant
	rs1410936	ILMN_1712347 (SFRS6)	distant
	rs6103330	ILMN_1712347 (SFRS6)	local
	rs13040414	ILMN_1712347 (SFRS6)	

Table S7. The average computational time (in seconds) of three methods for a fixed set of hyperparameters without the SNP screening with ten replicates for each simulation scenario. SNPs are only from chromosome 1.

Scenario	#Causal SNPs	Method	Parameter Tuning Time	Model Fitting Time	Total Time
weak-dense	60	FastLORS	164.85	4.34	169.19
		LORSEN	323.60	1.94	325.54
		LORS	164.54	12.63	177.18
	200	FastLORS	365.90	13.56	379.46
		LORSEN	317.87	2.31	320.17
		LORS	354.94	30.34	385.28
	400	FastLORS	456.50	21.63	478.14
		LORSEN	333.03	1.66	334.68
		LORS	456.38	42.56	498.94
strong-sparse	60	FastLORS	107.02	7.57	114.59
		LORSEN	390.63	2.02	392.66
		LORS	111.89	11.20	123.09
	200	FastLORS	167.97	13.39	181.36
		LORSEN	345.35	1.47	346.83
		LORS	168.06	19.94	188.00
	400	FastLORS	199.79	20.07	219.85
		LORSEN	306.73	2.15	308.88
		LORS	199.82	25.80	225.63

2 SUPPLEMENTARY FIGURES

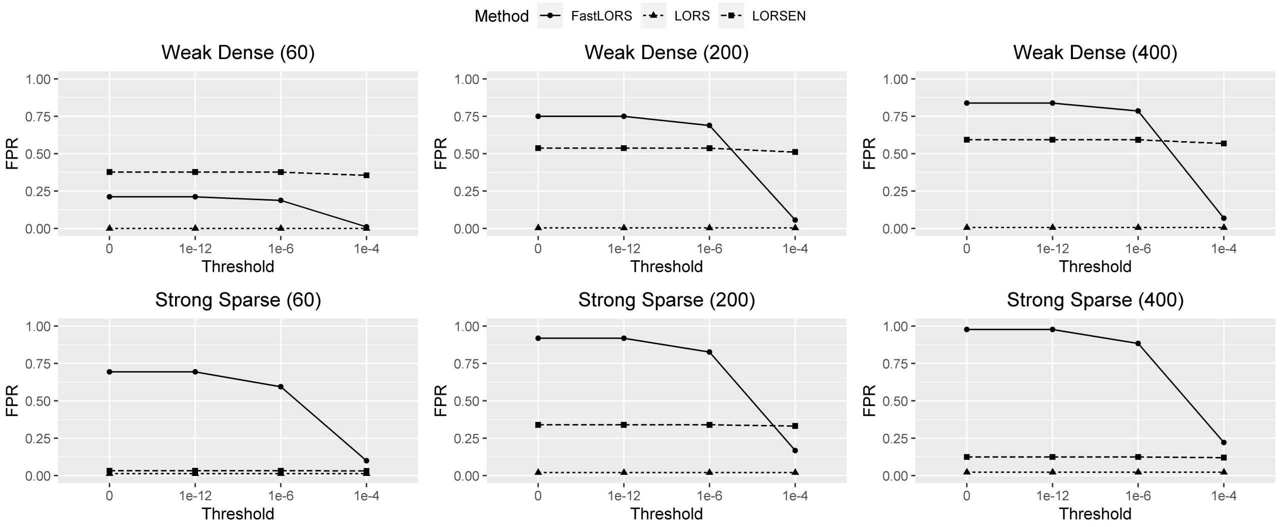

Figure S1: The average FPR of three methods with four different thresholds for the regression coefficients. All SNPs from chromosome 1 without the SNP screening were used. All the causal variants had the positive effects. For each simulation scenario, ten replicates were used.

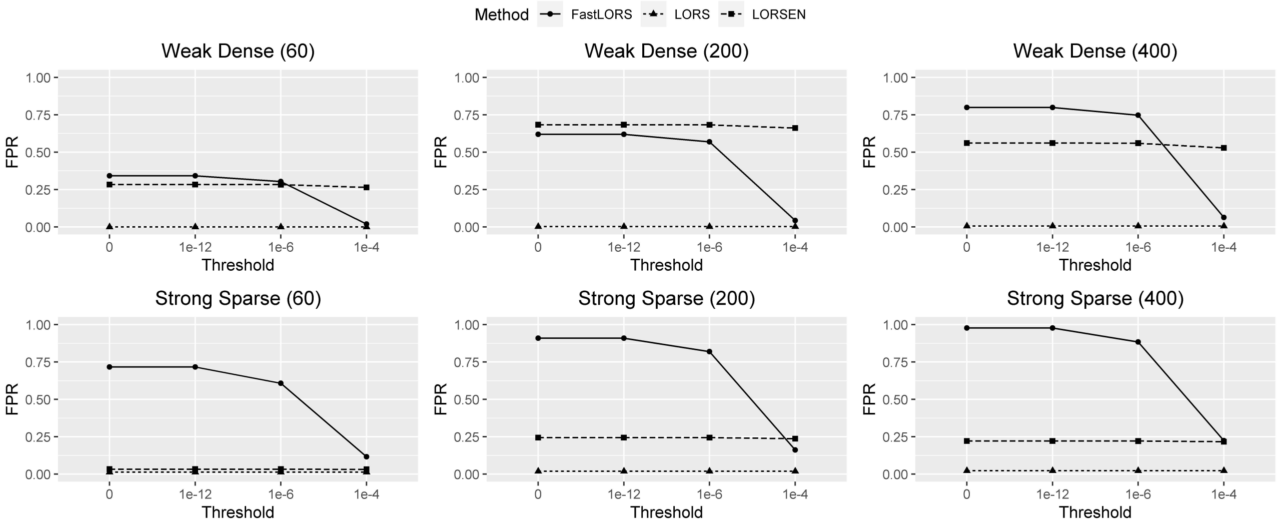

Figure S2: The average FPR of three methods with four different thresholds for the regression coefficients. All SNPs from chromosomes 1 and 21 without the SNP screening were used. All the causal variants had the positive effects. For each simulation scenario, ten replicates were used.

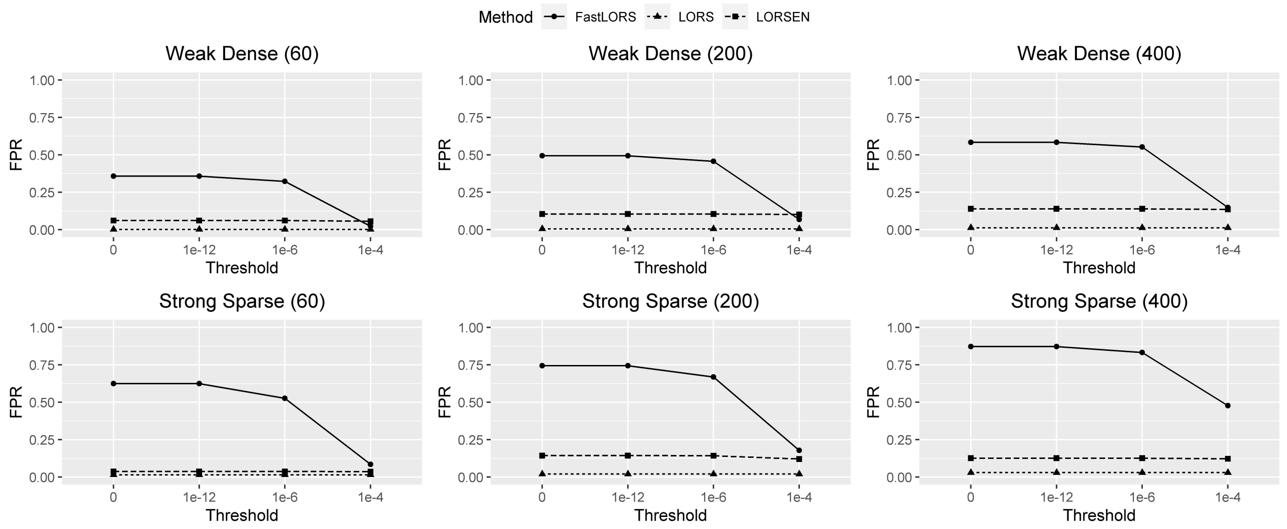

Figure S3: The average FPR of three methods with four different thresholds for the regression coefficients. All SNPs from chromosome 1 without the SNP screening were used. The half of causal variants had the positive effects while the other half of causal variants had the negative effects. For each simulation scenario, ten replicates were used.
