# Supplementary material for: LORSEN: Fast and Efficient eQTL Mapping With Low Rank Penalized Regression
Source: Front Genet. 2021 Nov 17;12:690926. doi: 10.3389/fgene.2021.690926 (PMC8636089; doi:10.3389/fgene.2021.690926)
Supplement: Supplementary file 3 [file Presentation3.pdf]

APPENDIX A

Lemma 1 For each $\tau \geqslant 0$ and $Y \in \mathbb{R}^{n_1 \times n_2}$, the solution of

$$\underset{X}{min} \quad \frac{1}{2}\|X - Y\|_F^2 + \tau\|X\|_* \tag{1}$$

is $S_\tau(Y) := US_\tau(\Sigma)V^T (= Prox_{\tau\|\cdot\|_*}(Y))$, where $S_\tau(\Sigma) = diag(\{(\sigma_i - \tau)_+\})$, $Y = U\Sigma V^T$, the singular value decomposition of matrix Y, $\Sigma = diag(\{\sigma_i\}_{1 \leqslant i \leqslant r})$, r is the rank of Y. $S_\tau(\cdot)$ is called singular value shrinkage operator.

Proof: see (Cai et al., 2010) or (Mazumder et al., 2010).

Lemma 2 For each fixed non-negative λ and $v \in \mathbb{R}^n$, the solution of

$$\underset{x}{min} \quad \frac{1}{2}\|x - v\|_2^2 + \frac{\lambda}{2}\|x\|_2^2 \tag{2}$$

is $(Prox_{\frac{\lambda}{2}\|\cdot\|_2^2}(v))_i = sign(v_i)(|v_i| - \lambda)_+$, $i = 1, 2, \cdots, n$, known as the (elementwise) soft thresholding operator.

Proof: see (Parikh and Boyd, 2014).

Lemma 3 For each fixed non-negative ρ and $v \in \mathbb{R}^n$, the solution of

$$\underset{x}{min} \quad \frac{1}{2}\|x - v\|_2^2 + \rho\|x\|_1 \tag{3}$$

is $Prox_{\rho\|\cdot\|_1}(v) = (1 - \frac{\rho}{max\{\|v\|_2, \rho\}})v$.

Proof: see (Parikh and Boyd, 2014).

Lemma 4 (soft-impute algorithm)

For the optimization problem

$$\begin{aligned} &\underset{X}{min} \quad \frac{1}{2}\|P_\Omega(Y - X)\|_F^2 + \tau\|X\|_* \\ &= \underset{X}{min} \quad \frac{1}{2}\|[P_\Omega(Y) + P_{\Omega^\perp}(X)] - X\|_F^2 + \tau\|X\|_*, \end{aligned}$$

the optimization solution can be obtained via updating X using $X \leftarrow S_\tau(P_\Omega(Y) + P_{\Omega^\perp}(X))$ with an arbitrary initialization.

Proof: see (Mazumder et al., 2010).

Theorem 1 A sufficient condition for $Prox_{f+g} = Prox_f \circ Prox_g$ is $\forall\ x \in \mathcal{H}$, $\partial g(Prox_f(x)) \supseteq \partial g(x)$, where $\mathcal{H}$ represents Hilbert space and $\circ$ represents composition of two operators.

Proof: see (Yu, 2013).

redDetails of Confidence Interval of AUC

red We followed the method used in (Hanley and McNeil, 1982) to calculate the 95% confidence interval (CI) of AUC. Let $\overline{\widehat{AUC}}$ and $Var(\widehat{AUC})$ denote the sample mean and the estimated variance of AUCs from ten replicates, respectively, the 95% CI of average AUC was calculated using the following formula:

$$\overline{\widehat{AUC}} \pm 1.96\sqrt{Var(\widehat{AUC})/10}. \tag{4}$$

red We used the following formula (Hanley and McNeil, 1982) to calculate $Var(\widehat{AUC})$:

$$Var(\widehat{AUC}) = \frac{q_0 + (n_1 - 1)q_1 + (n_2 - 1)q_2}{n_1 n_2}, \tag{5}$$

where $q_0 = \widehat{AUC}(1 - \widehat{AUC})$, $q_1 = \frac{\widehat{AUC}}{2 - \widehat{AUC}} - \widehat{AUC}^2$, $q_2 = \frac{2\widehat{AUC}^2}{1 + \widehat{AUC}} - \widehat{AUC}^2$, n_1 is the number of true positives, and n_2 is the number of true negatives.

References

Cai, J., Candès, E., and Shen, Z. (2010). A singular value thresholding algorithm for matrix completion. *SIAM J. Optim.*, 20:1956–1982.

Hanley, J. A. and McNeil, B. J. (1982). The meaning and use of the area under a receiver operating characteristic (roc) curve. *Radiology*, 143(1):29–36.

Mazumder, R., Hastie, T., and Tibshirani, R. (2010). Spectral regularization algorithms for learning large incomplete matrices. *The Journal of Machine Learning Research*, 11:2287–2322.

Parikh, N. and Boyd, S. (2014). Proximal algorithms. *Foundations and Trends in optimization*, 1(3):127–239.

Yu, Y.-L. (2013). On decomposing the proximal map. *Advances in neural information processing systems*, 26:91–99.
